# SARS-CoV-2 outbreaks in hospitals and long-term care facilities in Germany: a national observational study

**DOI:** 10.1016/j.lanepe.2021.100303

**Published:** 2022-01-14

**Authors:** Beneditta Suwono, Annika Steffen, Birgitta Schweickert, Viktoria Schönfeld, Michael Brandl, Mirco Sandfort, Niklas Willrich, Tim Eckmanns, Sebastian Haller

**Affiliations:** a Robert Koch Institute, Department for Infectious Disease Epidemiology, Berlin, Germany; b European Centre for Disease Prevention and Control, Stockholm, Sweden

## Abstract

**Background:**

Outbreaks of coronavirus disease (COVID-19) in hospitals and long-term care facilities (LTCFs) pose serious public health threats. We analysed how frequency and size of SARS-CoV-2 outbreaks in hospitals and LTCFs have altered since the beginning of the pandemic, in particular since the start of the vaccination campaign.

**Methods:**

We used mandatory notification data on SARS-CoV-2 cases in Germany and stratified by outbreak cases in hospitals and LTCFs. German vaccination coverage data were analysed. We studied the association of the occurrence of SARS-CoV-2 outbreaks and outbreak cases with SARS-CoV-2 cases in Germany throughout the four pandemic waves. We built also counterfactual scenarios with the first pandemic wave as the baseline.

**Findings:**

By 21 September 2021, there were 4,147,387 SARS-CoV-2 notified cases since March 2020. About 20% of these cases were reported as being related to an outbreak, with 1% of the cases in hospitals and 4% in LTCFs. The median number of outbreak cases in the different phases was smaller (≤5) in hospitals than in LTCFs (>10). In the first and second pandemic waves, we observed strong associations in both facility types between SARS-CoV-2 outbreak cases and total number of notified SARS-CoV-2 cases. However, during the third pandemic wave we observed a decline in outbreak cases in both facility types and only a weak association between outbreak cases and all cases.

**Interpretation:**

The vaccination campaign and non-pharmaceutical interventions have been able to protect vulnerable risk groups in hospitals and LTCFs.

**Funding:**

No specific funding

**Research in context**

**Evidence before this study**

The SARS-CoV-2 pandemic is placing a burden to healthcare facilities, particularly to hospitals and LTCFs. Non-pharmaceutical interventions have been aimed at protecting vulnerable risk groups since the beginning of the pandemic. Despite the strict implementation of these interventions, SARS-CoV-2 outbreaks still occurred in hospitals and LTCFs, with LTCFs being particularly hard hit in Germany during the second wave of pandemic. In the final weeks of the second pandemic wave, a vaccination campaign was introduced in Germany starting from 27 December 2020, targeting firstly vulnerable at-risk group such as residents of LTCFs and health care workers. Shortly after the start of the vaccination campaign, the number of SARS-CoV-2 outbreak cases in hospitals and LTCFs decreased sharply. It is therefore important to analyse to what extent the non-pharmaceutical and pharmaceutical interventions have influenced the number of SARS-CoV-2 outbreaks and their respective cases in hospitals and LTCFs. This will enable better infection and prevention control and pandemic preparedness in the future. For the purpose of this study, all PCR-confirmed SARS-CoV-2 cases from the German mandatory notification system were included. Data on vaccination coverage of nursing home residents (65 years and older) was originated from the national COVID-19 vaccination campaign database (DIM).

**Added value of this study**

The number of SARS-CoV-2 outbreaks and their respective cases in LTCFs were notably higher than in hospitals. Although the complex healthcare contact networks pose a particular challenge for infection prevention and control, hospitals in Germany could better control the number and size of outbreaks than LTCFs throughout the pandemic. The number of SARS-CoV-2 cases in the general population was strongly associated with the number of outbreaks and outbreak cases in both facilities in the first and second pandemic waves. However, these associations changed with the implementation of non-pharmaceutical, especially after introduction of pharmaceutical interventions shortly before third pandemic wave. Based on the counterfactual scenario, non-pharmaceutical and pharmaceutical interventions could have prevented up to 40% of outbreak cases in hospitals and 20% of outbreak cases in LTCFs for the second pandemic wave. This could be partly due to the fact that hospitals have better infection and prevention control capacity than in LTCFs.

**Implications of all the available evidence**

Targeted vaccination programs for healthcare workers and LTCF residents, as well as non-pharmaceutical interventions, have been able to stabilize the situation in hospitals and LTCFs. Although we observed a sharp decrease in outbreak cases in the third pandemic wave, these cases increased again slightly in the beginning of the fourth pandemic wave, nine months after the start of the vaccination campaign. This suggests that strict implementation of infection prevention and control (IPC) and non-pharmaceutical interventions will continue to hold a crucial role, especially in LTCFs, where the residents belong to one of the most vulnerable groups in the German population during the SARS-CoV-2 pandemic. Until vaccination against SARS-CoV-2 was available, the SARS-CoV-2 outbreaks in the hospitals and LTCFs may be best protected by low SARS-CoV-2 incidence in the general population.

Alt-text: Unlabelled box

## Introduction

Healthcare-associated SARS-CoV-2 outbreaks have been documented since the start of the pandemic and shown to amplify local outbreaks.^1^, ^2^, ^3^ Within hospitals and long-term care facilities (LTCFs), infections spread disproportionately among the elderly and vulnerable at-risk populations and often result in particularly severe patient outcomes.^4^ Outbreak control measures bind notoriously understaffed personnel in the healthcare sector. Concurrently the personnel are at-risk for becoming infected or having to stay in quarantine after exposure to SARS-CoV-2 infected persons. Thus, comprehensive infection prevention and control (IPC) measures were recommended in spring 2020 for all healthcare facilities and LTCFs including a mask mandate for personnel and patients.^5^ On December 27 the vaccination campaign started in Germany and prioritized at-risk groups, especially elderly (80+ years), LTCF residents, LTCF staffs, and healthcare workers with high risk of SARS-CoV-2 exposure or regular contact to vulnerable patients (CoronaImpfV^6^). Throughout the pandemic IPC measures, testing strategy and contact tracing were continuously optimised and adapted to the local situation.

In this study, we analysed differences in SARS-CoV-2 outbreaks in hospitals and LTCFs from March 2020 (CW 9/2020) to September 2021 (CW 37/2021). We aimed to compare the SARS-CoV-2 outbreaks in hospitals and LTCFs throughout the pandemic waves in Germany. We further analysed the association between the numbers of SARS-CoV-2 outbreaks and their respective cases in both healthcare facilities and SARS-CoV-2 cases in the general population before and after the implementation of non-pharmaceutical interventions (NPIs) and pharmaceutical intervention (PI). Lastly, we built up counterfactual scenarios on SARS-CoV-2 outbreaks and their respective cases by using the first pandemic wave as a baseline to estimate the numbers of patients and LTCF residents which were able to be protected throughout the other three pandemic waves.

## Methods

In this observational study, we analysed the German notification data and included all PCR-confirmed SARS-CoV-2 cases^7^ between March 2020 (Calendar Week 9; CW 9/2020) and September 2021 (CW 37/2021). We compared four different pandemic waves of SARS-CoV-2 infections in Germany: first wave (CW 9/2020 to 28/2020), second wave (29/2020–5/2021), third wave (6/2021–25/2021) and fourth wave (26/2021-37/2021).

An outbreak was defined when clusters of at least 2 PCR-confirmed SARS-CoV-2 infections were notified by the local public health authorities. Outbreaks were allocated to the calendar weeks according to the notification date of the index case of each outbreak.

**SARS-CoV-2 hospital outbreaks** were defined as outbreaks in hospitals. All outbreak cases related to health care workers (HCW) were included if epidemiologically linked to hospitals.

**SARS-CoV-2 LTCF-outbreaks** were defined when the outbreak setting was documented as LTCF.^8^ This included outbreak cases denoted as LTCF staffs.

We used Pearson correlation to study the correlation between the number of SARS-CoV-2 cases in the general population and the occurrence of outbreaks and outbreak cases. We would like to test whether the number of reported SARS-CoV-2 cases in the general population per calendar week was associated with the weekly number of reported SARS-CoV-2 hospital or LTCF outbreaks and/or reported SARS-CoV-2 outbreak cases. The correlation coefficients (R) aimed to study the variations in the outcome (SARS-CoV-2 outbreaks or outbreak cases), which could be explained by the predictor (SARS-CoV-2 cases in the general population). Additionally, we built the linear regression models to estimate association between the outcomes and the predictors for the four different pandemic waves. The 95% confidence intervals were calculated for the estimated slopes and intercepts. The model included the number of weekly cases, the pandemic waves and the interaction between weekly cases and the pandemic waves as predictors. The first wave was selected as a reference category for the model. This allowed the estimation of individual regression lines for each wave, but also to assess differences between the slopes for different waves. As the incubation period may last up to two weeks, consecutive cases in outbreaks occur later. Thus, SARS-CoV-2 outbreak cases were predicted based on the weekly cases in the general population lagged by two weeks.

To assess whether vaccination coverage was associated with the number and size of outbreaks, we used individual-level data from 14 of 16 federal states, representing 90% of the total German population. Precisely, we computed the cumulative uptake of full vaccination (2^nd^ dose) by calendar week for all individuals aged ≥65 years with the indication “nursing care home residents”. COVID-19 vaccination-coverage data from the central database of the COVID-19 vaccination campaign (DIM, *Digitales Impfquotenmonitoring*) from 27^th^ December 2020 (CW 53/2020) to 18^th^ April (CW 15/2021) was available.^9^ Until the end of March 2021 (CW 13/2021), vaccinations were exclusively carried out in state-authorized vaccination centres, hospitals, and via mobile vaccination teams. Since the beginning of April 2021 (CW 14/2021), vaccinations were also administered by general practitioners from whom we only received aggregated numbers of vaccinated individuals, that could not be used for our analyses of association. After CW 15/2021 (April 2021), health care workers could no longer be identified in the reported data as other professions were added to the indication for vaccination. Surveys carried out among the general population and among hospital staff were used to estimate vaccination coverage among health care workers.^10^^,^^11^ We used again Pearson correlation to further study the association between the number of LTCF outbreaks or outbreak cases and cumulative uptake of full vacination in LTCFs. We defined fully vaccinated as two weeks after the 2^nd^ dose and thus correlation between the vaccination coverage with outbreak cases lagged by two weeks.

Finally, we analysed counterfactual scenarios by estimating hypothetical numbers of SARS-CoV-2 outbreaks and their respective cases. For this analysis, we extrapolated the number of weekly outbreaks and outbreak cases from the weekly cases in the general population based on the coefficients for the linear model fitted for the first pandemic wave (baseline). Based on the estimated slope and intercept of the linear model for the first wave, we predicted counterfactual numbers of weekly outbreaks and outbreak cases based on the case numbers in the general population of the 3 other waves. In the counterfactual scenario and under our model assumptions, these predictions were interpreted as the numbers of outbreaks or outbreak cases that may have been prevented due to the NPIs and vaccination campaign (PI). These were calculated for every calendar week. To test the robustness of the first pandemic wave as baseline, we conducted additionally the same scenario with the second pandemic wave as baseline (sensitivity analyses).

All analyses were conducted in R (R Version x64 4.0.3).

### Ethics statement

Only anonymised patient data was analysed. For analyses of surveillance data from mandatory notification an ethical statement is not necessary according to German law (German Infection Protection Act).

### Role of the funding source

This study was internally funded by Robert Koch Institute. The Robert Koch Institute is the German National Public Health Institute under the portfolio of the German Federal Ministry of Health. The funders of the study had no role in study design, data collection, data analysis, data interpretation, or writing of the report.

## Results

In total, 4,147,387 SARS-CoV-2 cases have been reported from March 2020 (CW 9/2020) to September 2021 (CW 37/2021) (Data status: 21 September 2021). Twenty percent of these cases (828,474 outbreak cases) belonged to distinct SARS-CoV-2 outbreaks in various settings. Among these 40,400 outbreak cases (1% of all cases) occurred in hospitals and 156,349 outbreak cases (4% of all cases) in LTCFs.

### SARS-CoV-2 hospital and LTCF outbreaks in four pandemic waves

In the first pandemic wave (CW 9/2020, April 2020 until CW 28/2020, July 2020), there were 456 outbreaks with 5,004 outbreak cases in hospitals (Median age= 52 years) (Table 1). Sixty-six percent of these outbreak cases (n=3,318 cases) were reported in women. The case fatality ratio (CFR) was 10.3% (n=515). In LTCFs, 746 outbreaks with 14,810 outbreak cases were reported (Median age= 85 years). As in hospitals, SARS-CoV-2 LTCF outbreak cases were by majority female (10,593 cases, 71.6%). Eighteen percent of these patients were hospitalized (n=2,685). A higher CFR (27.5%, n=2,691 deaths) was observed in LTCF than in hospitals (*p* < 0.05). Following the first wave, measures to protect healthcare facilities and LTCFs from outbreaks were further optimized, including change of the testing strategies with expansion of test indications, laboratory testing capacities and screenings, and the recommendation to wear masks for both personnel and patients or LTCF residents. Visits from patients and LTCF residents were strongly regulated and suspended in case of outbreaks.

**Table 1:** SARS-CoV-2 outbreaks and outbreak cases in hospitals and long-term care facilities (LTCFs) in the four different pandemic waves. Data status: 21 September 2021. Y.o. = years old.

Variables	First wave (CW 9/2020, March 2020 - CW 28/2020, July 2020)	Second wave (CW 29/2020, July 2020 - CW 5/2021, February 2021)	Third wave (CW 6/2021, February 2021 - CW 25/2021, June 2021)	Fourth wave (CW 26/2021, June 2021 - CW 37/2021, September 2021)
Total number of SARS-CoV-2 cases	199,057	2,086,858	1,439,209	422,263
**Hospitals (SARS-CoV-2 hospital outbreaks)**				
Number of outbreaks (outbreak cases)	456 (5,004)	2,391 (29,365)	937 (5,639)	108 (393)
Proportion of outbreak cases in total cases %	2.5	1.4	0.4	0.1
Median outbreak size (range outbreak cases)	5 (2 - 197)	5 (2 - 351)	4 (2 - 82)	3 (2 - 15)
Median age (IQR)*	52 (34 – 75)	57 (36 – 79)	59 (39 – 79)	47 (31 – 68)
Outbreak cases in men (%)	1,683 (33.7)	10,074 (34.5)	2,250 (40)	158 (40.3)
Outbreak cases in women (%)	3,318 (66.3)	19,145 (65.5)	3,376 (60)	234 (59.7)
Deaths in outbreak cases (%)⁎⁎	515 (10.3)	3,414 (11.6)	672 (11.9)	7 (4)⁎⁎
**Long-term care facilities (SARS-CoV-2 LTCF outbreaks)**				
Number of outbreaks (cases)	746 (14,810)	4,466 (130,353)	846 (9,221)	196 (1,966)
Outbreak cases in elderly (≥65 y.o) (%)	9,803 (66.2)	91,322 (70.1)	5,690 (61.7)	1,389 (70.7)
Proportion of outbreak cases in total cases %	7.4	6.2	0.6	0.5
Median outbreak size (range outbreak cases)	13 (2 - 187)	21 (2 - 237)	7 (2 - 73)	8 (1- 50)⁎⁎⁎
Median age (IQR)	85 (80 – 90)	85 (80 – 90)	85 (80 – 90)	86 (81 – 91)
Outbreak cases in men (%)	4,211 (28.4)	36,316 (27.9)	2,627 (28.5)	493 (25.5)
Outbreak cases in women (%)	10,593 (71.6)	93,634 (72.1)	6,580 (71.5)	1,441 (74.5)
Outbreak cases with hospitalization (%)⁎⁎⁎⁎	2,685 (18.1)	14,154 (10.9)	1,025 (11.1)	244 (12.4)
Deaths in elderly outbreak cases (≥65 y.o.) (%)⁎⁎	2,691 (27.5)	19,305 (21.1)	914 (16.1)	99 (16.4)⁎⁎

⁎ Health care workers are still included. The median age should therefore be interpreted with caution.

⁎⁎ Deaths in outbreak cases is presented only until CW 34/2021 (August 2021).

⁎⁎⁎ The outbreak was notified in CW 37/2021 (September 2021) with minimum case of 1.

⁎⁎⁎⁎ The hospitalization rates with the complete information on status of hospitalization as denominator were observed with similar decreasing trends.

In the second pandemic wave (CW 29/2020, July 2021 until CW 05/2021, February 2021) 2,391 outbreaks (29,365 cases) were notified from hospitals and 4,466 outbreaks (130,353 cases) from LTCFs (Table 1). In comparison to the first pandemic wave, the outbreak size in hospitals remained stable (Median= 5 outbreak cases/outbreak), while it increased in LTCFs (Median= 21 outbreak cases/outbreak). Nevertheless, the proportion of hospitalizations (from 18.1% to 10.9%) and CFR (from 27.5% to 21.1%) in LTCFs decreased and the CFR in hospitals slightly increased from 10.3% to 11.6% (Table 1).

**Figure 1:**
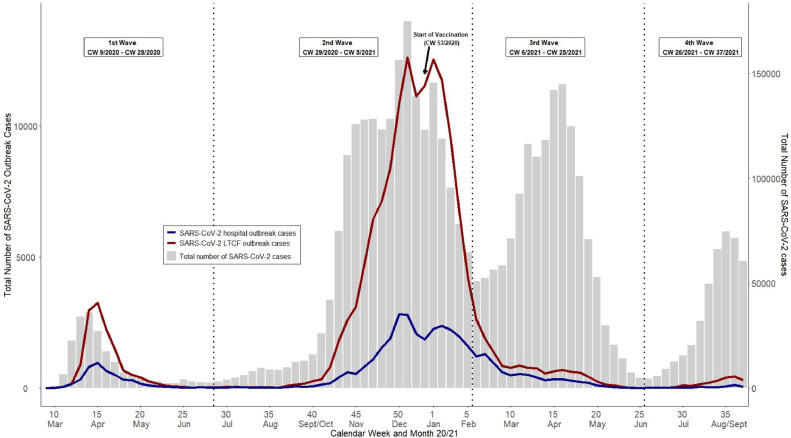
SARS-CoV-2 cases and SARS-CoV-2 outbreak cases in hospitals and long-term care facilities (LTCFs) per calendar week (CW) from CW 9/2020 (March 2020) until CW 37/2021 (September 2021) in Germany. Total SARS-CoV-2 cases (grey bars) are plotted with the SARS-CoV-2 outbreak cases in LTCF (red line) and hospitals (blue line). Outbreak cases were allocated to the calendar week of the reporting date of the outbreak's first reported case. X-axis: Calendar Week, left y-axis: total number of SARS-CoV-2 outbreak cases, right y-axis: total number of SARS-CoV-2 cases.

On CW 45/2020 (partial lockdown, 2 November 2020) and CW 51/2020 (second national lockdown, 16 December 2020) comprehensive contact reductions were introduced in Germany followed by the start of the vaccination campaign on 27 December 2020 (CW 53/2020).^12^ As vaccine availability was limited in the beginning, vaccinations had to be prioritised to at-risk individuals and healthcare workers. Thereafter the weekly numbers of outbreak cases decreased continuously, whereas the total number of SARS-CoV-2 cases decreased only until CW 6/2021 (February 2021) (Fig 1). In the third pandemic wave (CW 06/2021, February 2021 to CW 25/2021, June 2021), the total number of SARS-CoV-2 cases among the general population was lower than in the second pandemic wave. This low total number of SARS-CoV-2 cases was accompanied by low numbers of SARS-CoV-2 outbreaks: 937 outbreaks (5,639 outbreak cases) in hospitals and 844 outbreaks (9,221 cases) in LTCFs (Table 1). The median outbreak size was smaller than during the first and second pandemic waves for hospitals (4 outbreak cases/outbreak) and LTCFs (7 outbreak cases/outbreak). The CFR decreased in LTCFs (16.1%) compared to the second pandemic wave, while in hospitals it remained stable (11.9%) (Table 1).

In the early period of the fourth pandemic wave, we have observed low numbers of outbreaks and outbreak cases.

### Association of SARS-CoV-2 outbreaks with SARS-CoV-2 cases in the general population

**Figure 2:**
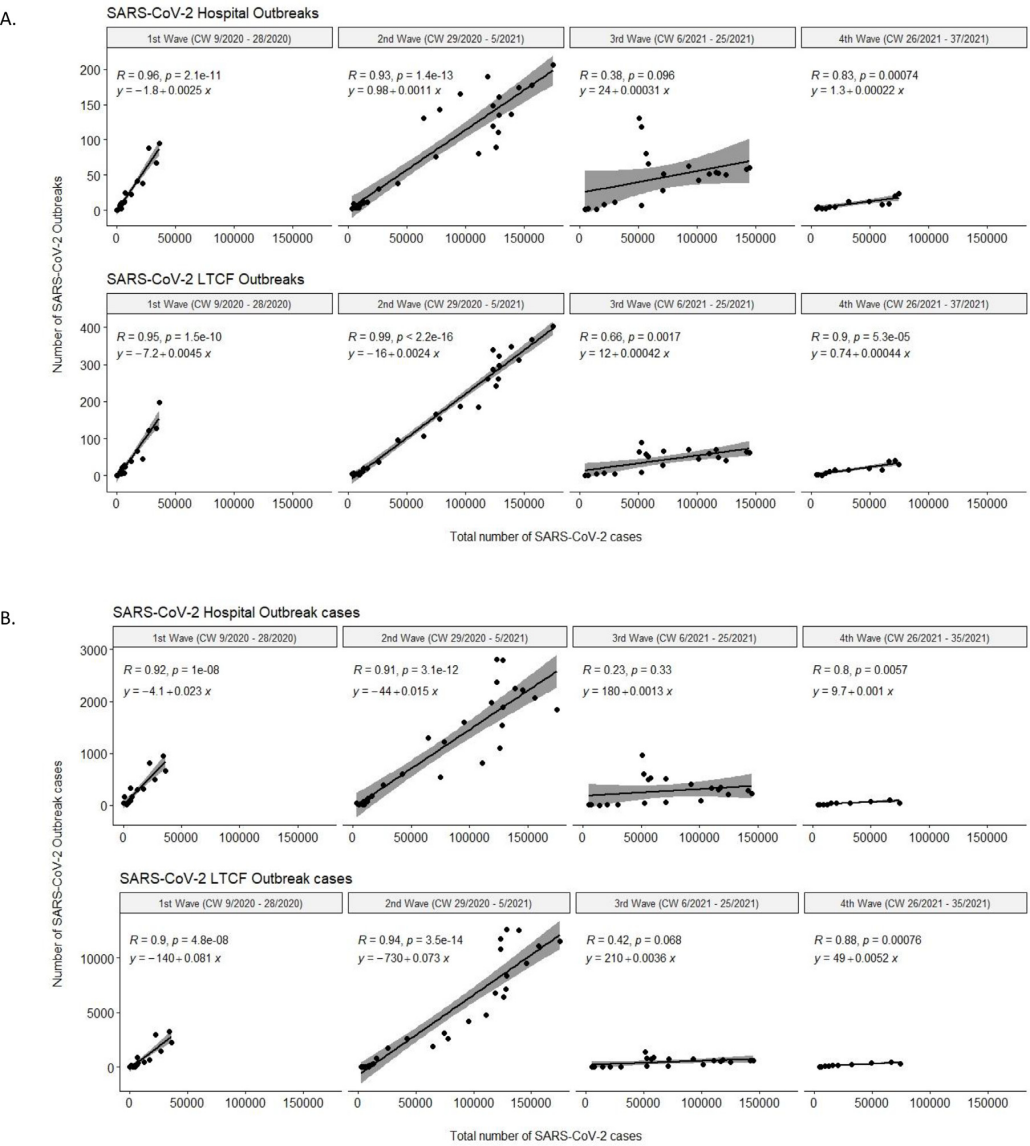
A) Correlation of weekly SARS-CoV-2 cases and SARS-CoV-2 outbreaks in hospitals and long-term care facilities (LTCFs) for the first wave (CW 9/2020, March 2020 – 28/2020, July 2020), second wave (CW29/2020, July 2020 – CW 5/2021, February 2021), third wave (CW 6/2021, February 2021 – CW 25/2021, June 2021) and fourth wave (CW 26/2021, June 2021 – CW 37/2021, September 2021). X-axis: total number of SARS-CoV-2 cases, Y-axis: number of SARS-CoV-2 outbreaks. Each point denotes weekly reported SARS-CoV-2 cases and SARS-CoV-2 outbreaks. The regression lines were presented with 95% confidence interval (CI). Correlations were calculated with Pearson correlation. B) Correlation of weekly SARS-CoV-2 cases and SARS-CoV-2 outbreaks cases two weeks after in hospitals and long-term care facilities (LTCFs). X-axis: total number of SARS-CoV-2 cases, Y-axis: number of SARS-CoV-2 outbreak cases. Each point denotes weekly reported SARS-CoV-2 cases and two weeks after SARS-CoV-2 outbreak cases. The regression lines were presented with 95% CI. Correlations were calculated with Pearson correlation.

During the first wave, weekly reported SARS-CoV-2 cases in the general population in Germany were significantly associated with weekly reported SARS-CoV-2 outbreaks in hospitals (R=0.96, p < 0.001) and LTCFs (R=0.95, p < 0.001) (Fig 2). Similar associations for both facility types were observed for weekly reported cases and outbreak cases that occurred two weeks later in the general population in Germany (hospitals R=0.92, p < 0.001and LTCF R=0.9, p < 0.001). Judging from the weekly increase, an additional 25 hospital outbreaks [CI 95% 15 – 35 outbreaks] and 45 LTCF outbreaks [CI 95% 37 – 53 outbreaks] were estimated for every 10,000 weekly reported cases. Furthermore, we calculated an increase of 234 hospital outbreak cases [CI 95% 115 – 353 outbreak cases] and 813 LTCF outbreak cases [CI 95% 375 – 1,251 outbreak cases] two weeks after a weekly increase of 10,000 cases (Table 2).

**Table 2:** Intercepts and slopes for the linear regression equations with the 95% CI for each pandemic wave.

SARS-CoV-2 outbreaks	First wave (CW 9/2020, ``March 2020 - CW 28/2020, July 2020)	Second wave (CW 29/2020, July 2020 - CW 5/2021, February 2021)	Third wave (CW 6/2021, February 2021 - CW 25/2021, June 2021)	Fourth wave (CW 26/2021, June 2021 - CW 37/2021, September 2021)
**SARS-CoV-2 hospital outbreaks**				
Intercept [95% CI]	-1.8 [-16.4; 12.9]	0.98 [-12.8; 14.8]	24.4 [3.5; 45.2]	1.3 [-22; 24.6]
Slopes [95% CI]	0.0025 [0.0015; 0.0035]	0.0011 [0.001; 0.0013]	0.0003 [0.0001; 0.0005]	0.0002 [-0.0003; 0.0007]
**SARS-CoV-2 LTCF outbreaks**				
Intercept [95% CI]	-7.2 [-19.3; 4.9]	-15.8 [-27.2; -4.4]	12.2 [-5.1; 29.4]	0.7 [-18.5; 20]
Slopes [95% CI]	0.0045 [0.0037; 0.0053]	0.0024 [0.0022; 0.0025]	0.0004 [0.0002; 0.0006]	0.0004 [0; 0.0009]

SARS-CoV-2 outbreak cases	First wave (CW 9/2020, March 2020 - CW 28/2020, July 2020)	Second wave (CW 29/2020, July 2020 - CW 5/2021, February 2021)	Third wave (CW 6/2021, February 2021 - CW 25/2021, June 2021)	Fourth wave (CW 26/2021, June 2021 - CW 37/2021, September 2021)
**SARS-CoV-2 hospital outbreak cases**				
Intercept [95% CI]	-4.1 [-180; 171.8]	-43.8 [-209.9; 122.3]	176.1 [-75.2; 427.5]	9.7 [-275.7; 295.1]
Slopes [95% CI]	0.0234 [0.0115; 0.0353]	0.015 [0.0132; 0.0169]	0.0013 [-0.0017; 0.0043]	0.001 [-0.0065; 0.0085]
**SARS-CoV-2 LTCF outbreak cases**				
Intercept [95% CI]	-138 [-786.3; 510.3]	-729.9 [-1,341.9; -117.8]	214.1 [-712.5; 1,140.6]	49.1 [-1,003; 1,101.2]
Slopes [95% CI]	0.0813 [0.0375; 0.1251]	0.0735 [0.0667; 0.0803]	0.0036 [-0.0074; 0.0146]	0.0052 [-0.0226; 0.0329]

In the second pandemic wave the weekly SARS-CoV-2 cases in the general population were again significantly associated with weekly numbers of SARS-CoV-2 outbreaks in hospitals (R=0.93, p < 0.001) and LTCFs (R=0.99, p < 0.001) (Fig 2). The estimated weekly increase of outbreaks in both facility types was lower than in the first pandemic wave; 11 hospital outbreaks [95% CI 10 - 13] and 24 LTCF outbreaks [95% CI 22 - 25] were expected for every increase of 10,000 weekly cases. As for outbreak cases, an increase by 150 hospital outbreak cases [95% CI 132 - 169] and 735 LTCF outbreak cases [95% CI 667 - 803] was expected two weeks after weekly increases of 10,000 cases (Table 2).

In the third pandemic wave, smaller correlation coefficients than correlation coefficients in the first and second waves of the weekly SARS-CoV-2 outbreaks and weekly SARS-CoV-2 cases in the general population were observed in hospitals (R=0.38, p < 0.01) and in LTCFs (R=0.66, p < 0.01) (Fig 2). These coefficients were also smaller for outbreak cases in both facility types (hospitals R=0.23, p>0.05 and LTCFs R=0.42, p>0.05). Moreover, the estimated weekly numbers of outbreaks (3 hospital outbreaks [95% CI 1 - 5] and 4 LTCF outbreaks [95% CI 2 - 6]) and outbreak cases two weeks later (13 hospital outbreak cases [95% CI 0 - 43] and 36 LTCF outbreak cases [95% CI 0 - 146]) by weekly increase of 10,000 cases were considerably smaller than in the previous waves (Table 2).

In the fourth pandemic wave, there were significant associations for both facilities (p > 0.05 for both facilities): with correlation coefficients in hospitals R=0.8 and LTCFs R=0.88 (Fig 2). The weekly estimated numbers of outbreaks were almost similar as estimated in second wave: 2 hospital outbreaks [95% CI 0 - 7] with 10 outbreak cases [95% CI 0 - 85] and 4 LTCF outbreaks [95% CI 0 - 9] with 52 outbreak cases [95% CI 0 - 329] (Table 2).

The linear regression models with the first pandemic wave as the baselines showed significant differences (p-value < 0.05) between the slopes of the first wave and the third and fourth pandemic wave, respectively (Supplementary File 1).

### Vaccination coverage and SARS-CoV-2 outbreaks

**Figure 3:**
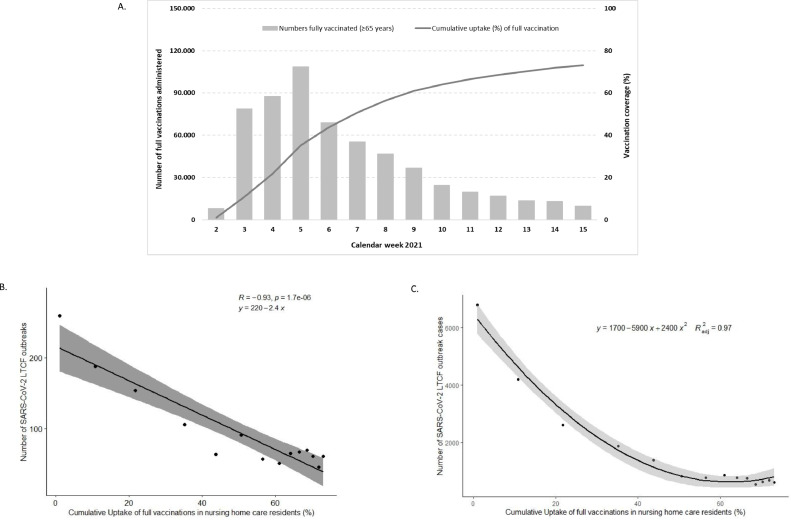
A) Number and cumulative uptake (%) of full vaccinations among individuals aged ≥65 years with indication “nursing home care residents”. It displays the weekly number of fully vaccinated individuals and the cumulative uptake of full vaccination among individuals aged ≥65 years who had the indication “nursing home residents”. X-axis: Calendar Week 2021, left Y-axis: number of full vaccinations administered (grey bars) and right Y-axis: vaccination coverage in percent (line). Correlation between the cumulative uptake (%) of full vaccinations and number of B) SARS-CoV-2 LTCF outbreaks and C) outbreak cases. X-axis: weekly cumulative uptake (%) in fourteen German Federal States and Y-axis: number of SARS-CoV-2 LTCF B) weekly outbreaks or C) outbreak cases two weeks after the vaccination. Each point denotes weekly reported SARS-CoV-2 outbreaks or outbreak cases. The regression lines were presented with 95% confidence interval (CI). Correlations were calculated with Pearson correlation.

The weekly number of completed vaccination series increased strongly from CW 2/2021 onwards and peaked in CW 5/2021 (January 2021), where the second wave ended. By CW 15/2021 (April 2021) there were 587,138 vaccinated individuals aged ≥65 in 14 German Federal States. The cumulative uptake of full vaccination increased to 73% in CW 15/2021 (April 2021) (Fig 3A). As for healthcare facilities, surveys indicate that by beginning of April around 77% to 83% of health care workers had received one, and 32% to 48% had received two doses of COVID-19 vaccine.^10^^,^^11^

The number of SARS-CoV-2 LTCF outbreaks and their respective cases decreased since the start of the vaccination campaign. The weekly numbers of SARS-CoV-2 LTCF outbreaks were negatively associated (R=-0.93, p < 0.001) with the increased cumulative uptake of full vaccinations among LTCFs individuals aged ≥ 65 years old (Fig 3B and 3C).

### What might have happened to SARS-CoV-2 outbreaks had there been no interventions? A counterfactual scenario

**Figure 4:**
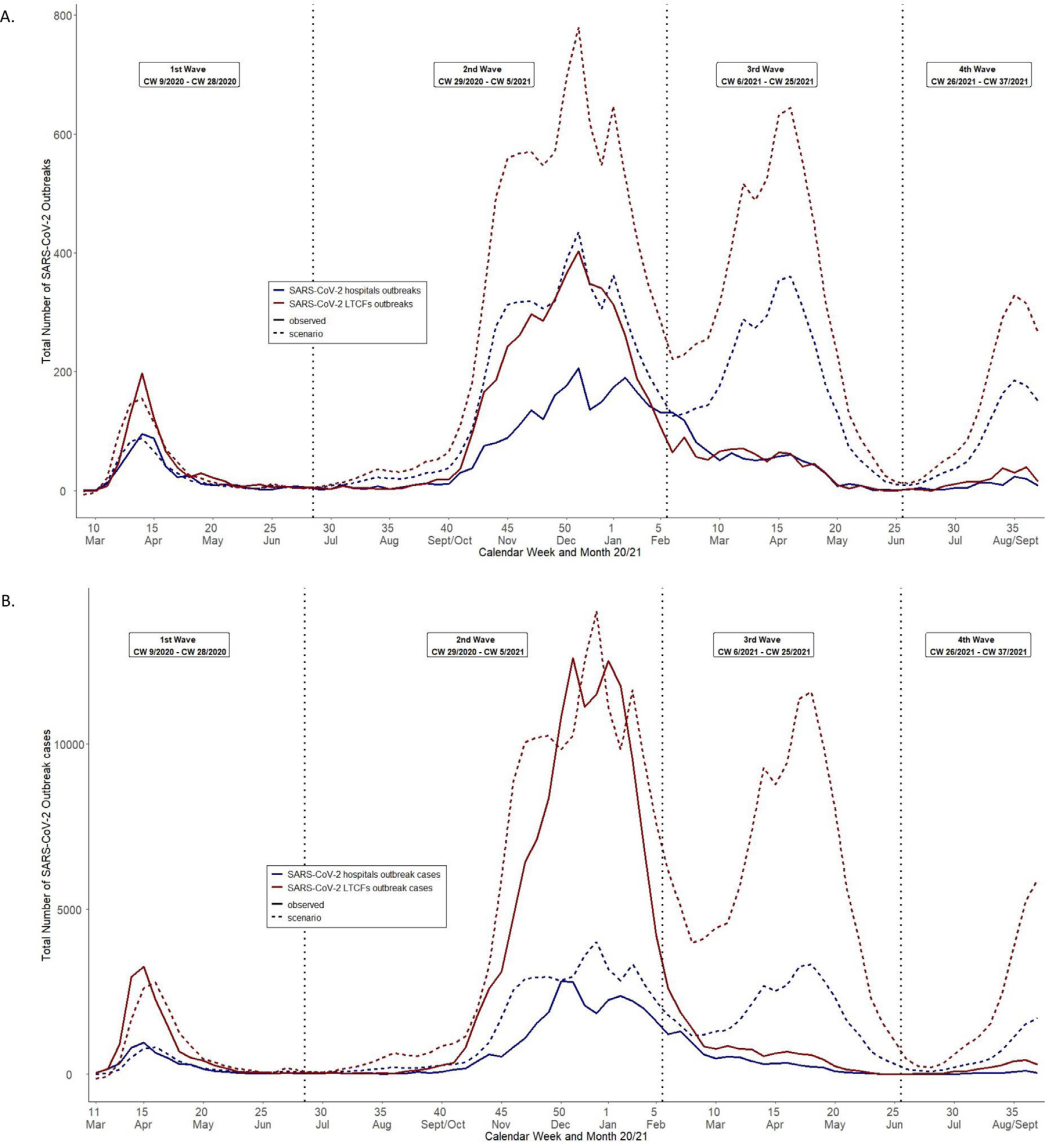
Counterfactual scenarios on A) number of SARS-CoV-2 hospital outbreaks (solid and dotted blue line) and SARS-CoV-2 LTCF outbreaks (solid and dotted red line) and B) SARS-CoV-2 hospital outbreak cases (solid and dotted blue line) and SARS-CoV-2 LTCF outbreak cases (solid and dotted red line) for four different pandemic waves. For B) the scenario of SARS-CoV-2 cases were estimated with the numbers of SARS-CoV-2 total cases from two weeks before (9/2020 – 35/2021). A) X-axis: calendar week from 9/2020 to 37/2021 and Y-axis: Number of SARS-CoV-2 outbreaks. B) X-axis: calendar week from 11/2020 to 37/2021 and Y-axis: Number of SARS-CoV-2 outbreak cases.

Assuming that we saw a “natural” association of cases in the general population and outbreaks and outbreak cases in hospitals and LTCFs during the first wave, when experiences with SARS-CoV-2 and targeted interventions were still limited, there were an estimated 2,706 SARS-CoV-2 hospital outbreaks with 18,683 outbreak cases and 4,657 SARS-CoV-2 LTCF outbreaks and 34,039 outbreak cases that were prevented in the second pandemic wave where NPIs were implemented (Table 3, Fig 4). One month after the vaccination campaign started, there were an estimated 2,579 SARS-CoV-2 hospital outbreaks with 28,184 outbreak cases and 5,450 SARS-CoV-2 LTCF outbreaks with 104,788 outbreak cases that were prevented. In the fourth pandemic wave, there were an estimated 913 SARS-CoV-2 hospital outbreaks with 6,358 outbreak cases and 1,607 SARS-CoV-2 LTCF outbreaks with 20,213 outbreak cases that were prevented.

**Table 3:** Counterfactual scenarios with 95% CI for second, third and fourth pandemic waves for number of outbreaks and their respective cases. The first wave was used as the references.

Total number of outbreaks	2nd Wave (CW 29/2020, July 2020 - 5/2021, February 2021)	3rd Wave (CW 6/2021, February 2021 - 25/2021, June 2021)	4th Wave (CW 26/2021, June 2021 - 37/2021, September 2021)
**SARS-CoV-2 hospital outbreaks**			
Observed	2,390	937	108
Scenario [95% CI]	5,096 [3,194; 6,997]	3,516 [2,253; 4,778]	1,021 [655; 1,387]
Difference between observed and scenario (prevented)	2,706	2,579	913
**SARS-CoV-2 LTCF outbreaks**			
Observed	4,465	846	196
Scenario [95% CI]	9,122 [7,547; 10,697]	6,296 [5,250; 7,341]	1,803 [1,500; 2,106]
Difference between observed and scenario (prevented)	4,657	5,450	1,607

Total number of outbreak cases	2nd Wave (CW 31/2020, July 2020 - 7/2021, February 2021)*	3rd Wave (CW 8/2021, February 2021 - 27/2021, July 2021)*	4th Wave (CW 28/2021, July 2021 - 37/2021, September 2021)*
**SARS-CoV-2 hospital outbreaks**			
Observed	30,026	5,411	387
Scenario [95% CI]	48,709 [25,824; 71,594]	33,595 [18,405; 48,786]	6,745 [3,677; 9,813]
Difference between observed and scenario (prevented)	18,683	28,184	6,358
**SARS-CoV-2 LTCF outbreaks**			
Observed	131,473	9,453	1,985
Scenario [95% CI]	165,512 [81,161; 249,864]	114,241 [58,250; 170,232]	22,198 [10,889; 33,506]
Difference between observed and scenario (prevented)	34,039	104,788	20,213

⁎ The observed outbreak cases were computed two weeks after the observed total SARS-CoV-2 cases in general population.

The counterfactual scenario using the second pandemic wave as baseline did not show any relevant difference on the prevented number of SARS-CoV-2 outbreaks or their respective cases for the third and fourth pandemic waves (Supplementary Table 2).

## Discussion

SARS-CoV-2 LTCF outbreaks were larger and more severe than SARS-CoV-2 hospital outbreaks in all four pandemic waves in Germany. The number of outbreaks in both types of facilities might be approximately similar, however the number of outbreak cases in LTCFs was larger than in hospitals. The lower median number of SARS-CoV-2 cases in subsequent outbreaks in hospitals indicates that if outbreaks occurred, they may have been efficiently stopped and that there was a learning curve throughout the pandemic. This was not the case for LTCFs, where the number of cases per outbreak increased and was less effectively affected by NPIs in the second pandemic wave. In the counterfactual scenarios, the scenario numbers of SARS-CoV-2 highlighted again the differences between outbreaks in hospitals and in LTCFs. Through the strict implementation of IPC measures in hospitals in the second wave, the factual numbers of SARS-CoV-2 infections may have been prevented by up to 40%. In LTCFs, the outbreak cases were approximately estimated as the observed outbreak cases in the second pandemic wave and only up to 20% were prevented. It showed that LTCFs had larger difficulties in controlling the outbreaks than hospitals, probably due to the challenging and insufficient IPC capacities.^8^ Moreover, the hospital staffs have received more intensive and comprehensive training on IPC in compare to personnel in LTFCs. Thus, better trained personnel and improvement of implementation of IPC measures in the LTCFs, which faced unique challenges due to the concept of communal living, may help to better protect LTCF residents from infectious disease outbreaks in the future.

We found more SARS-CoV-2 outbreak cases in women than men at both types of facilities. This finding was different from the proportions in the general population in Germany, which suggested similar proportions of men and women.^12^ However, this finding was supported by previous findings in SARS-CoV-2 LTCF outbreaks, where most LTCF residents were about two-thirds female.^1^ When it comes to the severity of illness, the proportion of SARS-CoV-2 infections in women were lower than men.^12^

We observed a strong decrease in numbers of SARS-CoV-2 infections, outbreaks and outbreak cases after the advent of NPIs in the second pandemic wave. The counterfactual scenarios suggested that a large number of SARS-CoV-2 outbreaks and to lesser extent respective cases could have been prevented alone with NPIs. At the beginning of the second pandemic wave, NPIs played an important role in reducing transmission, as there were no pharmaceutical interventions available for COVID-19.^5^ These interventions have also been able to support hospitals^13^ in maintaining strict IPC measures during the SARS-CoV-2 outbreaks.

NPIs were more effective when combined with vaccination (PI).^14^^,^^15^ Our study highlighted a sharp decrease of SARS-CoV-2 outbreak cases in the third pandemic wave after the start of vaccination campaign. During the third pandemic wave, the number of SARS-CoV-2 outbreaks and outbreak cases showed a smaller increase than expected in relation to the rising incidence of SARS-CoV-2-cases in the general population. For both types of facilities, a decline of the CFR could be observed. Moreover, the numbers of prevented SARS-CoV-2 outbreaks and their respective cases in both facility types were larger than in the second pandemic wave. This suggests that the vaccination is able to protect the vulnerable at-risk groups within healthcare facilities especially in LTCFs. Reduced transmission of SARS-CoV-2 cases in LTCFs has been also reported in Spain^16^ and Canada.^17^ In Germany, considerable reductions in COVID-19 incidence, hospitalisation and mortality among individuals aged ≥80 years have been observed in parallel with increasing vaccination coverage, providing first evidence on the success of the immunization campaign.^18^ Moreover, this decrease was also accompanied by the highest numbers of testing among the group of vaccinated 80 years old.^19^ Improved case management and medical treatment may have led to reduction of hospitalisation and CFR in SARS-CoV-2 infected patients. Until recently, the effect of these measures on transmission of SARS-CoV-2 in the healthcare setting appears to be limited.

Some argued that high risk populations could be protected from SARS-CoV-2 infection alone by strict control measures for these populations. We here present evidence, that after introduction of targeted interventions, the spread of COVID-19 within the facilities could not be adequately contained. Occurrence of outbreaks strongly associated with SARS-CoV-2 incidence in the general population^20^^,^^21^, suggesting that individuals in institutions could not fully be protected from infection. Further one may ask, what role visitors play in the introduction of SARS-CoV-2 into facilities. Even though evidence was not sufficient to recommend a stop of patient visits and of LTCF residents, visitors were restricted in many facilities during the pandemic. Nonetheless, the number of new outbreaks remained relatively stable, suggesting that SARS-CoV-2 is introduced mainly through staff.^22^^,^^23^

This is the first study to examine the alteration in the frequency and case numbers of SARS-CoV-2 outbreaks in hospitals and LTCFs in Germany since the beginning of the pandemic during the nine months of the immunization campaign. By using data from the mandatory notification system, this study was able to describe a nationwide situation of SARS-CoV-2 outbreaks in hospitals and LTCFs. For the purpose of this study, we defined specific time periods reflecting the time course of the four pandemic waves, which were different from those previously described.^1^^,^^12^^,^^24^^,^^25^ However, this did not have a major impact on the analyses as we only separated the number of summer months in which few SARS-CoV-2 infections were reported.

Although we analysed the data from German mandatory notification system, the proportions of hospitalized cases might be under represented due to incomplete information. The outbreaks occurred frequently over long period of time. During the pandemic there were massive workloads that were carried by local public health authorities and this could lead to incomplete data entries. Moreover, testing capacities and modalities changed throughout the pandemic in Germany. During the second and third waves in particular age groups >65 were tested regularly and the test incidence was highest among the age group above 80 years. For screening antigen tests were also conducted in LTCFs, however positive results were generally confirmed with PCR. Thus, underreporting in these age groups may have been lower than in others. Overall the under-detection factor of SARS-CoV-2 cases in the German mandatory surveillance system was estimated to be 1.8.^26^ Moreover, this study only considered the association between the cumulative uptake of full vaccination in nursing care home residents with the number of SARS-CoV-2 outbreaks and their respective cases. Although healthcare workers might play specific role in the SARS-CoV-2 outbreaks in hospitals and LTCFs, we could not assess the vaccination coverage among personnel.

We did not consider any confounders in this study such as improvement of testing capacities throughout the pandemic waves^1^, the role of novel variant of concern (VOC) and the seasonality. The seasonality has been frequently associated with respiratory diseases foremost influenza. It might also play a role in low transmission of SARS-CoV-2 in summer.^27^^,^^28^ Causality may not be inferred from this ecological study. The study findings should therefore be interpreted with caution.

In summary, our analysis shows that outbreak frequency and size may be affected by NPIs, which were in particular effective in hospitals. However, until the targeted vaccination program for SARS-CoV-2 was introduced, the number of outbreaks associated with SARS-CoV-2 incidence. A combination of nonpharmaceutical interventions with a targeted vaccination program has possibly prevented more than 150,000 SARS-CoV-2 infections in outbreaks in LTCFs and more than 50,000 SARS-CoV-2 infections in outbreaks in hospitals throughout the pandemic. Nine months after the start of vaccination campaign, however, healthcare-associated SARS-CoV-2 outbreaks are still being reported. Against the background of waning immune protection after vaccination, the value of continuing NPIs in these high-risk settings will rise.

## Declaration of interests

All authors have disclosed that there is no conflict of interest.

## Appendix Supplementary materials

Image, application 1
