# Supplementary material for: SARS-CoV-2 outbreaks in hospitals and long-term care facilities in Germany: a national observational study
Source: Lancet Reg Health Eur. 2022 Jan 14;14:100303. doi: 10.1016/j.lanepe.2021.100303 (PMC8759004; doi:10.1016/j.lanepe.2021.100303)
Supplement: Supplementary file 1 [file mmc1.docx]

**Supplementary Files**

**Supplementary Table 1. Linear regression model with the interaction terms for the outbreaks and their respective cases in hospitals and LTCFs.**

| **Variables** | **SARS-CoV-2 outbreaks** | | **SARS-CoV-2 outbreak cases** | |
| --- | --- | --- | --- | --- |
|  | **Estimation  [95% CI]** | **p-value** | **Estimation  [95% CI]** | **p-value** |
| **SARS-CoV-2 hospital outbreaks** | | | | |
| Intercept | -1.7547  [-16.3621; 12.8527] | 0.81 | -4.0961  [-179.9819; 171.7898] | 0.96 |
| Weekly SARS-CoV-2 cases in the general population (per case) | 0.0025  [0.0015; 0.0035] | 4.1 x 10^-6^ | 0.0234  [0.0115; 0.0353] | 1.9 x 10^-4^ |
| *Pandemic wave* | | | | |
| First wave | **Ref** | | **Ref** | |
| Second wave | 2.735  [-17.3544; 22.8243] | 0.79 | -39.7093  [-281.6025; 202.1838] | 0.74 |
| Third wave | 26.1248  [0.6446; 51.6051] | 0.045 | 180.2323  [-126.5722; 487.0368] | 0.25 |
| Fourth wave | 3.0546  [-24.4203; 30.5295] | 0.83 | 13.7916  [-321.487; 349.0702] | 0.94 |
| *Interaction between pandemic wave and cases per week* | | | | |
| First wave | **Ref** | | **Ref** | |
| Second wave | -0.0013  [-0.0023; -0.0003] | 9.5 x 10^-3^ | -0.0084  [-0.0204; 0.0036] | 0.17 |
| Third wave | -0.0022  [-0.0032; -0.0011] | 6.9 x 10^-5^ | -0.0221 [-0.0343; -0.0098] | 6.0 x 10^-4^ |
| Fourth wave | -0.0022  [-0.0034; -0.0011] | 1.5 x 10^-4^ | -0.0224  [-0.0365; -0.0083] | 2.2 x 10^-3^ |
| **SARS-CoV-2 LTCF outbreaks** | | |  |  |
| Intercept | -7.2411  [-19.3415; 4.8592] | 0.24 | -137.968  [-786.264; 510.328] | 0.67 |
| Weekly SARS-CoV-2 cases in the general population (per case) | 0.0045  [0.0037; 0.0053] | 4.7 x 10^-17^ | 0.0813 [0.0375; 0.1251] | 4.2 x 10^-4^ |
| *Pandemic wave* | | | | |
| First wave | **Ref** | | **Ref** | |
| Second wave | -8.5552  [-25.1966; 8.0862] | 0.31 | -591.8955  [-1483.4873; 299.6963] | 0.19 |
| Third wave | 19.3926  [-1.7145; 40.4997] | 0.071 | 352.046  [-778.8019; 1482.8939] | 0.54 |
| Fourth wave | 7.9851  [-14.7743; 30.7445] | 0.49 | 187.0738  [-1048.7264; 1422.874] | 0.76 |
| *Interaction between pandemic wave and cases per week* | | | | |
| First wave | **Ref** | | **Ref** | |
| Second wave | -0.0021  [-0.0029; -0.0013] | 2.7 x 10^-6^ | -0.0078  [-0.0521; 0.0365] | 0.73 |
| Third wave | -0.0041  [-0.0049; -0.0032] | 1.3 x 10^-14^ | -0.0777  [-0.1229; -0.0325] | 1.0 x 10^-3^ |
| Fourth wave | -0.004  [-0.005; -0.0031] | 7.4 x 10^-13^ | -0.0761  [-0.128; -0.0243] | 4.6 x 10^-3^ |

**Supplementary Table 2. Counterfactual Scenario with 95% CI with the second pandemic wave as baseline.**

| **Total number of outbreaks** | **3rd Wave  (CW 6/2021, February 2021 - 25/2021, June 2021)** | **4th Wave  (CW 26/2021, June 2021 - 37/2021, September 2021)** |
| --- | --- | --- |
| **SARS-CoV-2 hospital outbreaks** | | |
| Observed | 937 | 108 |
| Scenario  [95% CI] | 1,648  [1,433; 1,862] | 489  [361; 618] |
| Difference between observed and scenario (prevented) | 711 | 381 |
| **SARS-CoV-2 LTCF outbreaks** | | |
| Observed | 846 | 196 |
| Scenario  [95% CI] | 3,090  [2,913; 3,268] | 810  [703; 916] |
| Difference between observed and scenario (prevented) | 2,244 | 614 |
| **Total number of outbreak cases** | **3rd Wave  (CW 8/2021, February 2021 - 27/2021, July 2021)*** | **4th Wave  (CW 28/2021, July 2021 - 37/2021, September 2021)*** |
| **SARS-CoV-2 hospital outbreaks** | | |
| Observed | 5,411 | 387 |
| Scenario  [95% CI] | 20,738  [18,157; 23,318] | 3,917  [2,582; 5,252] |
| Difference between observed and scenario (prevented) | 15,327 | 3,530 |
| **SARS-CoV-2 LTCF outbreaks** | | |
| Observed | 9,453 | 1,985 |
| Scenario  [95% CI] | 91,174  [81,662; 100,686] | 14,016  [9,095; 18,937] |
| Difference between observed and scenario (prevented) | 81,721 | 12,031 |
